# Determining Whether Sex and Zygosity Modulates the Association between APOE4 and Psychosis in a Neuropathologically-Confirmed Alzheimer’s Disease Cohort

**DOI:** 10.3390/brainsci12091266

**Published:** 2022-09-19

**Authors:** Mila Valcic, Marc A. Khoury, Julia Kim, Luis Fornazzari, Nathan W. Churchill, Zahinoor Ismail, Vincenzo De Luca, Debby Tsuang, Tom A. Schweizer, David G. Munoz, Corinne E. Fischer

**Affiliations:** 1 Keenan Research Centre for Biomedical Science, Li Ka Shing Knowledge Institute, St. Michael’s Hospital, 209 Victoria Street, Toronto, ON M5B 1T8, Canada; mila.valcic@unityhealth.to (M.V.); marc.khoury@unityhealth.to (M.A.K.); jjuliakim@gmail.com (J.K.); luis.fornazzari@gmail.com (L.F.); nchurchill.research@gmail.com (N.W.C.); tom.schweizer@unityhealth.to (T.A.S.); david.munoz@unityhealth.to (D.G.M.); 2 Department of Neurology, Faculty of Medicine, University of Toronto, Toronto, ON M5S 3H2, Canada; 3 Departments of Psychiatry, Clinical Neurosciences, Community Health Sciences, and Pathology, Hotchkiss Brain Institute and O’Brien Institute of Public Health, University of Calgary, Calgary, AB T2N 4Z6, Canada; ismailz@ucalgary.ca; 4 Division of Geriatric Psychiatry, Centre for Addiction & Mental Health, Toronto, ON M5T 1R8, Canada; vincenzo.deluca@unityhealth.to; 5 GRECC, VA Puget Sound and Department of Psychiatry and Behavioral Sciences, University of Washington, Seattle, WA 98195-6560, USA; dwt1@uw.edu; 6 Institute of Medical Sciences, University of Toronto, Toronto, ON M5S 1A8, Canada; 7 Institute of Biomaterials and Biomedical Engineering, University of Toronto, Toronto, ON M5S 3G9, Canada; 8 Division of Neurosurgery, Department of Surgery, Faculty of Medicine, University of Toronto, Toronto, ON M5T 1P5, Canada; 9 Division of Neurosurgery, St. Michael’s Hospital, Toronto, ON M5B 1W8, Canada; 10 Department of Laboratory Medicine and Pathobiology, University of Toronto, Toronto, ON M5S 1A8, Canada; 11 Division of Pathology, St. Michael’s Hospital, Toronto, ON M5B 1W8, Canada; 12 Department of Psychiatry, Faculty of Medicine, University of Toronto, Toronto, ON M5T 1R8, Canada

**Keywords:** Alzheimer’s disease, Lewy body, APOE, psychosis, neuropsychiatric

## Abstract

Background: The APOE4 allele is a genetic risk factor for developing late-onset Alzheimer’s disease (AD). Previous work by our group revealed that female APOE4 homozygotes with Lewy body (LB) pathology were more likely to experience psychosis compared to female APOE4 non-carriers, whereas in males there was no APOE4 dose-dependent significant effect. The objective of this study was to refine our previous findings by adjusting for covariates and determining the probability of an APOE4 sex-mediated effect on psychosis. Methods: Neuropathologically confirmed AD patients with LB pathology (n = 491) and without LB pathology (n = 716) were extracted from the National Alzheimer’s Coordinating Center (NACC). Patients were classified as psychotic if they scored positively for delusions and/or hallucinations on the Neuropsychiatric Inventory. Analysis consisted of a preliminary unadjusted binary logistic regression and a Generalized Additive binary logistic regression Model (GAM) to predict the relationship between APOE4 status and sex on the presence of psychosis in both cohorts, adjusting for age, education and MMSE. Results: In the cohort with LB pathology, female APOE4 homozygotes were significantly more likely to experience psychosis compared to female APOE4 non-carriers (OR = 4.15, 95%CI [1.21, 14.2], *p* = 0.023). Female heterozygotes were also more likely to experience psychosis compared to female APOE4 non-carriers, but to a lesser extent (OR = 2.37, 95%CI [1.01, 5.59], *p* = 0.048). There was no significant difference in males with LB pathology or in any sex in the cohort without LB pathology. Conclusions: Sex and zygosity influence the effect of APOE4 on psychosis in neuropathologically confirmed AD patients, with the effect being limited to females with LB pathology.

## 1. Introduction

The Apolipoprotein (APOE) gene has been linked to the risk and age of onset of Alzheimer’s Disease (AD). The APOE protein has three main isoforms: APOE2, APOE3 and APOE4, which are encoded by the APOE ε2, ε3, and ε4 alleles, respectively [1]. While the ε2 allele is considered protective and the ε3 allele is the most common variant in the general population, the ε4 allele is a genetic risk factor for AD. Individuals with the APOE4 allele have a significantly increased risk of developing late-onset AD compared to individuals with ε2 or ε3 alleles. Those with a single APOE4 allele have a 2- to 4-fold greater risk, whereas individuals with two copies of the APOE4 allele have an 8- to 12-fold greater risk of developing AD [1]. The mechanism(s) by which APOE4 increases the risk of developing AD is not entirely understood; however, there is strong evidence to suggest that APOE4 increases amyloid pathology [2] and that it may exacerbate tau pathology [3]. Past research has shown that when the expression of astrocytic APOE4 is increased during the seeding stage of amyloid development, amyloid deposition and neuritic dystrophy are enhanced [2]. The same was not found when increasing the expression of astrocytic APOE3. Additionally, they found that APOE4, but not APOE3, increased the amyloid-beta half-life and increased amyloid-related gliosis in the brain. These findings suggest that APOE4 impacts amyloid-beta clearance and increases amyloid-beta aggregation in the brain. Furthermore, using cerebral organoids, one study has shown that APOE4 worsens tau pathology in both healthy subjects and AD patients [3]. These findings strongly support the hypothesis that APOE4 is involved in AD neuropathology; however, this effect appears to be more pervasive in females compared to males [4]. Previous work has revealed a significant effect of APOE4 and sex interaction on CSF t-tau and *p*-tau in individuals with mild cognitive impairment; specifically, females experience greater APOE4-mediated tauopathy compared to males [4].

AD patients exhibit a wide range of symptoms, including impairments in memory and cognition [5]. Psychotic symptoms, which include delusions and hallucinations, are also common in AD patients, occurring in approximately 50% of patients [6,7,8]. The most common delusions include delusions of persecution, infidelity, abandonment and/or believing that deceased individuals are alive, whereas the most common hallucinations are visual hallucinations. AD patients with psychosis demonstrate increasingly rapid cognitive decline and more cortical synaptic loss compared to AD patients without psychosis, suggesting a more severe phenotype of the disease [6] and thus an importance for further research in this area [7,8,9].

The link between APOE4 and psychosis in AD has been previously researched, with relatively mixed findings, likely due to varying sample sizes [6]. An early study conducted in 1996 revealed that AD patients with an APOE 3/4 genotype had a more than three-fold increase in signs of psychosis compared to controls or AD patients with an APOE 3/3 genotype [10]. In contrast, a study in 2006 found no significant association between the APOE genotype and the presence of delusions or hallucinations [11]. It has been noted that familial aggregation and heritability studies have been critical in determining that genetic variation likely influences the risk of psychosis in AD, with some studies estimating the heritability of psychosis in AD as high as 61% [12]. Previous work by our group examining a cohort of neuropathologically confirmed AD patients found that female APOE4 homozygotes with Lewy body (LB) pathology were more likely to experience psychosis compared to female APOE4 non-carriers, whereas in males there was no dose-dependent difference [13]. In females without LB pathology, having one or two copies of the APOE4 allele was not associated with psychosis. LB pathology is an additional neuropathological lesion found in approximately half of autopsy-confirmed AD patients [14]. Research has shown that APOE4 exacerbates the activity and neurotoxicity of α-synuclein, the primary constituent of LB pathology [15]. The objective of this study was to refine our previous findings by additionally adjusting for covariates (age, education, MMSE scores) and comparing neuropsychiatric symptom profiles between at-risk and not-at-risk females. We hypothesized that after adjusting for covariates, female APOE4 homozygotes with LB pathology would still be more likely to experience psychosis compared to female APOE4 non-carriers.

## 2. Materials and Methods

### 2.1. Data Source

**Figure 1:**
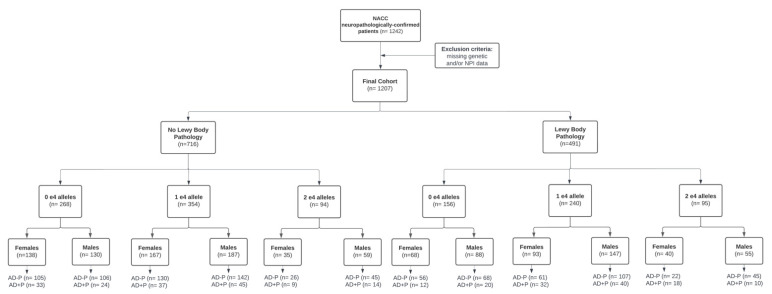
Cohort Breakdown.

The patient cohort was obtained from the National Alzheimer’s Coordinating Center (NACC) database. Two datasets were utilized: the Uniform Data Set (UDS) and the Neuropathology Data Set (NP). The UDS consists of data collected annually for each subject, including demographics, neurological examination findings, diagnosis, etc. The NP data set is composed of autopsy records for a subset of patients from the UDS and the Minimum Data Set (MDS). Demographics collected for this study’s analyses included age, sex and education. Cognitive status was assessed using the Mini Mental State Examination (MMSE), and the presence of psychotic symptoms was assessed based on the Neuropsychiatric Inventory Questionnaire (NPI-Q), which evaluated delusions and/or hallucinations in the month prior to their first clinical visit. Using the same cohort of patients included in the study by Kim et al., data were gathered strictly in individuals with NIA-AA Reagan high probability of AD, determined through both frequent neuritic plaques on CERAD and Braak Stage of V or VI on post-mortem neuropathological evaluation [13,16], who had available genetic and NPI data (n = 1207). Figure 1 presents a breakdown of the cohort and exclusion criteria.

Breakdown of the patient sample utilized in this study. Patients with neuropathologically confirmed AD were stratified based on Lewy body pathology (presence or absence), APOE4 status, and further by sex and psychosis status (AD-P: no psychosis, AD+P: psychotic).

### 2.2. Statistical Analyses

The Statistical Package for the Social Sciences (IBM SPSS Statistics for Mac, Version 28.0) and R-Studio were used for the statistical tests. Two cohorts within the dataset were extracted: patients with LB pathology (LB (+)) and those without LB pathology (LB (+)). Within each cohort, patients were classified as E4 (−), E4 or E44 based on the number of APOE ε4 alleles they carried (0, 1, 2, respectively) and stratified by sex. All analyses were performed in both LB (+) and LB (−) cohorts separately; *p* values ≤ 0.05 were considered statistically significant, and odds ratios were estimated at a 95%CI.

A preliminary binary logistic regression was utilized to determine if the presence of psychosis could be predicted based on APOE4 status and sex, without adjusting for covariates. Next, the primary analysis consisted of a Generalized Additive Binary Logistic Regression Model (GAM) for each cohort to determine the relationship between psychosis and sex-specific zygosity while adjusting for covariates. Here, GAM was implemented in R-studio using the Mixed GAM Computation Vehicle with Automatic Smoothness Estimation (MGCV) package [17], serving as an approach to model the non-linear relationship between a binary outcome variable and its continuous predictors. For all GAM models, age, education and MMSE scores were controlled for as covariates and modeled with thin plate smoothing splines, removing assumptions of linearity. 

A post-hoc analysis was conducted to examine probability estimates for the symptom severity of all 12 neuropsychiatric domains on the NPI between the at-risk female group (APOE4 homozygotes with LB pathology and psychosis) and females who did not meet the at-risk criteria. Additionally, the relationship between risk status (0 = non-risk group, 1 = at-risk group) and the presence of the other neuropsychiatric domains included in the NPI was evaluated using a Chi-square test or Fisher’s exact test, as appropriate. Lastly, the relationship between risk status and the type of psychotic profile that patients exhibit (only delusions, only hallucinations, or both delusions and hallucinations) was analyzed.

## 3. Results

### 3.1. Descriptive Statistics

**Figure 2:**
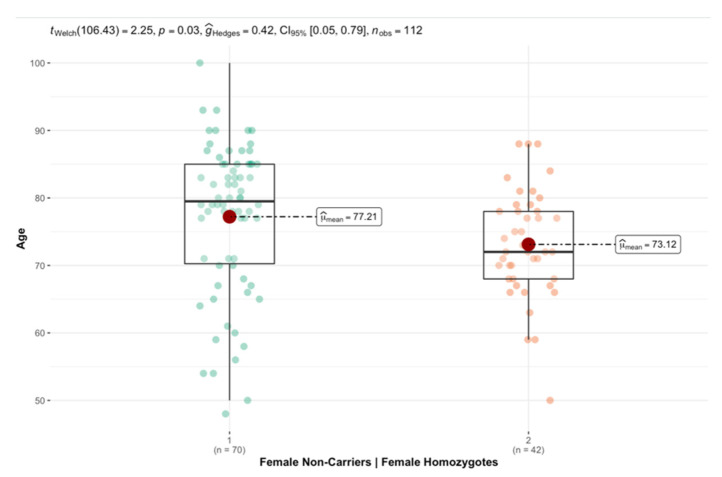
Average age x, female APOE4 non-carriers vs. female APOE4 homozygotes.

Table 1 provides a summary of the general descriptive statistics for APOE4 status. As illustrated in Figure 2, female APOE4 homozygotes were, on average, significantly younger than female APOE4 non-carriers (*p* = 0.03). This trend was not observed in males.

Summary of descriptive statistics in neuropathologically-confirmed AD patients, stratified by APOE4 status (E4 (−) = 0 APOE4 alleles, E4 = 1 APOE4 allele, E44 = 2 APOE4 alleles).

Graphical depiction of the average age of female APOE4 non-carriers and female APOE4 homozygotes, illustrating the significant difference between the two groups. On average, female APOE4 homozygotes were significantly younger than female APOE4 non-carriers (g = 0.42, 95%CI [0.05, 0.79], *p* = 0.03).

### 3.2. LB (+) Cohort

Unadjusted Results. In the LB (+) cohort, 26.9% of the patients had psychosis (n = 132). Female APOE4 homozygotes were 3.8 times more likely to experience psychosis compared to female APOE4 non-carriers (OR = 3.8, 95%CI [1.6, 9.2], *p* = 0.003), and 3.7 times more likely compared to male APOE4 homozygotes (OR = 3.7, 95%CI [1.5, 9.3], *p* = 0.006), in the unadjusted model. Within the cohort of patients who were male with LB pathology and the presence of psychosis, 14.3% were APOE4 homozygous (n = 10). In contrast, in the cohort of female patients with LB pathology and the presence of psychosis, 29% were APOE4 homozygous (n = 18), approximately double.

**Figure 3:**
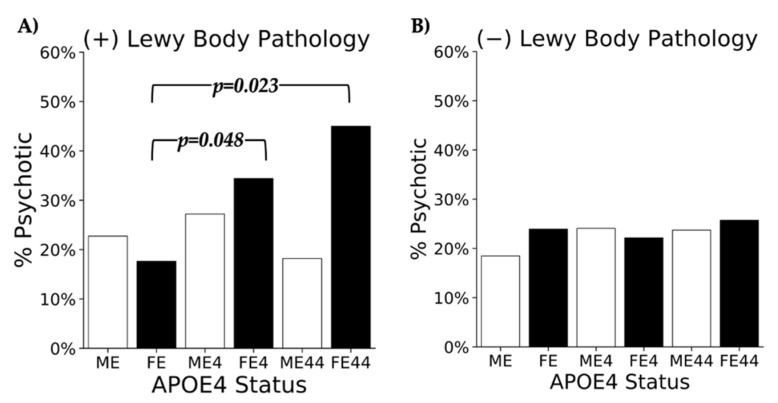
% Psychotic Across Cohorts.

GAM Results. Results from the GAM model, after adjusting for covariates, revealed that female APOE4 homozygotes were 4.15 times more likely to experience psychosis compared to female APOE4 non-carriers (OR = 4.15, 95%CI [1.21, 14.2], *p* = 0.023). Female APOE4 heterozygotes were 2.37 times more likely compared to female APOE4 non-carriers (OR = 2.37, 95%CI [1.01, 5.59], *p* = 0.048). In contrast, male homozygote and heterozygote carriers displayed no significant association with the presence of psychosis when compared to male non-carriers. All of the group comparisons that were analyzed using the GAM model in the LB (+) cohort are shown in Table 2. Regarding covariates, MMSE scores differed significantly for all group comparisons, whereas education scores were only associated with female homozygotes when compared to non-carriers. Age did not reveal any significant associations with group comparisons. Figure 3A displays the percent of individuals psychotic, stratified by sex and APOE4 status within the LB (+) cohort. Of note, 45% of female heterozygotes with LB pathology had psychosis.

GAM Binary logistic regression analysis within the LB (+) cohort, examining the relationship between APOE4 status and the presence of psychosis across sex while adjusting for age, education and MMSE.

Graphical representation of the % psychotic across sex and APOE4 status: ME (male non-carriers), FE (female non-carriers), ME4 (male APOE4 heterozygotes), FE4 (female APOE4 heterozygotes), ME44 (male APOE4 homozygotes), and FE44 (female APOE4 homozygotes) in the (**A**) LB (+) and (**B**) LB (−) cohorts. In the LB (+) cohort, after adjusting for covariates (age, education, MMSE scores), FE44 was significantly more likely to experience psychosis compared to FE (*p* = 0.023), and FE4 was significantly more likely to experience psychosis compared to FE (*p* = 0.048). There was no statistically significant association between APOE4 and the presence of psychosis in males. There was no statistically significant association between APOE4 and the presence of psychosis in either sex in the LB (−) cohort.

### 3.3. LB (−) Cohort

In the LB (−) group, 22.6% of the patients had psychosis (n = 162). There was no statistically significant association between APOE4 and the presence of psychosis in either sex. All of the group comparisons that were analyzed using the GAM model in the LB (−) cohort are shown in Table 3. Regarding covariates, MMSE scores differed significantly for all but one group comparison, whereas age did not appear to play a significant role. Figure 3B displays the percent of individuals psychotic, stratified by sex and APOE4 status within the LB (−) cohort.

GAM Binary logistic regression analysis within the LB (−) cohort, examining the relationship between APOE4 status and the presence of psychosis across sex while adjusting for age, education and MMSE.

### 3.4. Post Hoc Analysis: Neuropsychiatric Symptoms

Results from the Chi-square and Fisher’s exact test analyses were non-significant, as risk status and psychotic profile and presence of each non-psychotic neuropsychiatric symptom did not yield significant *p*-values: agitation (*p* = 0.084), depression (*p* = 0.289), anxiety (*p* = 0.082), elation (*p* = 0.457), apathy (*p* = 0.690), disinhibition (*p* = 0.780), irritability (*p* = 0.637), motor disturbance (*p* = 0.300), nighttime behaviors (*p* = 0.991), and appetite (*p* = 0.797). While the association between risk status and the type of psychotic profile patients exhibited was not statistically significant (*p* = 0.252), the at-risk group showed a slight trend of being more likely to experience only delusions. When analyzing the association between risk status and probability of exhibiting each NPI symptom severity, individuals with a risk status of 0 showed a trend of having mild symptoms, whereas individuals with a risk status of 1 showed a trend of having severe symptoms for the NPI domains anxiety, apathy, disinhibition and nighttime behaviors. However, these findings were not statistically significant and therefore no conclusions can be drawn. Interestingly, there was no significant association between risk status and the severity of psychotic symptoms (delusions and hallucinations).

## 4. Discussion

Previous studies examining the role of APOE4 in psychosis in AD have produced varied findings, with genetic association studies not finding a significant association between APOE and psychosis [12]. This has been attributed to many factors, one of which is the sample size. Across 23 studies examining the association between APOE4 and psychosis in AD, the median sample size was 173 patients [12]. A strength of the present study is the large sample size of 1214 patients. Previous work using the NACC database found no association of the APOE4 allele with psychosis in UDS [18]. They analyzed the association of APOE4 with psychosis in a cohort of participants with probable AD and possible AD diagnosed through clinical assessments. They found that neither APOE4 carrier status nor number of APOE4 alleles were associated with psychosis. The findings of the present study, using the NP data set, reveal that there is an association between carrying 1 and 2 copies of the APOE4 allele and psychosis in females with a high load of both components of AD pathology, determined through post-mortem neuropathological evaluation. Our findings suggest that, when controlling for age, education and cognitive status, sex and zygosity moderate the effect of APOE4 on psychosis in a cohort of neuropathologically confirmed AD patients. Specifically, the effect of APOE4 on the presence of psychosis is limited to females with LB pathology and strongest in homozygotes. The significant effect found in female APOE4 heterozygotes with LB pathology compared to female APOE4 non-carriers with LB pathology on the presence of psychosis is novel to this paper. In previous analyses, which were not adjusted for covariates, this effect was not revealed [13]. Interestingly, only 14.2% of males with LB pathology and psychosis were APOE4 homozygous, whereas approximately double, 29%, of females with LB pathology and psychosis were APOE4 homozygous. These findings suggest that in female AD patients, genetics, specifically APOE4 dosage, is a factor that drives psychosis symptoms in those with LB pathology, whereas the effect of APOE4 on psychosis is much more attenuated in male AD patients with LB pathology. It would be of interest to conduct future neurobiological studies to examine what other factors predict psychosis in males with neuropathologically confirmed AD. 

The findings of the present study are clinically significant, as they reveal a cohort of AD patients that appear to be particularly vulnerable to developing psychosis, which is associated with several comorbidities, such as depression and increased mortality [6,7,8]. Further study of this subgroup of patients may reveal the underlying mechanisms of psychosis in AD, potentially through the formation of Lewy bodies. Previous work has revealed a significant association between the APOE ε4 allele and the presence of concomitant Lewy bodies in AD [19]. Additionally, Kim et al. demonstrated that two copies of the APOE4 allele were positively associated with the formation of Lewy bodies in females [13]. The present study did not find a significant association between APOE4 and sex in psychosis in AD patients without LB pathology; therefore, LB formation may be a key factor in the underlying mechanism of psychosis among female APOE4 carriers and should be further explored.

While the primary findings of this study suggest that females who are APOE4 homozygous with LB pathology are significantly more likely to experience psychosis; interestingly, the post-hoc analyses suggest this at-risk group is not more likely to experience additional neuropsychiatric symptoms and does not appear to have more severe psychotic symptoms. Future studies with larger sample sizes are required to examine these relationships further. It is important to note that the sample size of the at-risk group was small (n = 18), and this may not be a true representation of this group’s behavior profile. Identifying neuropsychiatric symptoms associated with the presence of psychosis in the at-risk group may help identify potential neuropsychiatric phenotypes that may have implications for treatment [20]. 

There are some limitations to this study. The NPI-Q was used to classify patients as psychotic, relying on symptoms in the month prior to the clinic visit. It is possible that patients who were classified as non-psychotic based on the NPI-Q have experienced psychosis greater than 1 month prior to their clinic visit, and thus were improperly classified. In addition, the findings of this study cannot determine causation, only associations. Finally, we relied on data collected across several Alzheimer’s Disease Research Centers (ADRCs). While data were collected using a standardized evaluation of patients enrolled in ADRC clinics, it is possible that there was some variation in data collection.

## 5. Conclusions

In conclusion, this study demonstrated that female APOE4 homozygotes with LB body pathology were significantly more likely to experience psychosis compared to female APOE4 non-carriers with LB pathology. Female APOE4 heterozygotes with LB body pathology were also significantly more likely to experience psychosis compared to female APOE4 non-carriers, but to a lesser extent. These findings suggest that the APOE4 allele dosage plays a role in psychosis in female AD patients, whereas the same association is not seen in males. Further studies should be conducted to determine what predicts psychosis in male AD patients, to determine if these female at-risk patients behave significantly differently across other neuropsychiatric domains and severity levels and to see if these findings extend to clinical data sets.

## Figures and Tables

**Table 1:** Descriptive Statistics.

		E4 (−) (n = 424)	E4 (n = 594)	E44 (n = 189)
Sex				
	Male	218 (51.4%)	334 (56.2%)	114 (60.3%)
	Female	206 (48.6%)	260 (43.8%)	75 (39.7%)
Lewy body pathology				
	LB (−)	268 (63.2%)	354 (59.6%)	94 (49.7%)
	Males	130 (59.6%)	187 (56.0%)	59 (51.8%)
	Females	138 (67.0%)	167 (64.2%)	35 (46.7%)
	LB (+)	156 (36.8%)	240 (40.4%)	95 (50.3%)
	Males	88 (40.4%)	147 (44.0%)	55 (48.2%)
	Females	68 (33.0%)	93 (35.8%)	40 (53.3%)
Age				
	Mean (SD)	75 (12)	74 (10)	73 (8)
	Males	73 (11)	74 (10)	73 (8)
	Females	77 (12)	75 (11)	73 (8)
	Median [Min, Max]	78 [36, 100]	76 [44, 98]	73 [44, 90]
	Males	76 [36, 96]	75 [47, 95]	73 [44, 90]
	Females	80 [38, 100]	77 [44, 98]	73 [50, 89]
MMSE				
	Mean (SD)	19 (8)	17 (9)	18 (8)
	Males	19 (8)	17 (9)	19 (7)
	Females	19 (8)	17 (8)	15 (8)
	Median [Min, Max]	21 [0, 30]	19 [0, 30]	19 [0, 30]
	Males	21 [0, 30]	19 [0, 30]	21 [0, 30]
	Females	20 [0, 30]	19 [0, 30]	16 [0, 30]
Education				
	Mean (SD)	15 (3)	15 (3)	16 (3)
	Males	16 (3)	16 (3)	16 (3)
	Females	14 (3)	14 (3)	15 (3)
	Median [Min, Max]	16 [2, 28]	16 [3, 25]	16 [8, 22]
	Males	16 [3, 28]	16 [6, 25]	16 [8, 22]
	Females	14 [2, 20]	14 [3, 20]	16 [8, 20]

**Table 2:** LB (+) Cohort, GAM.

Females	Covariates (*p*-value)		
Female Homozygotes vs. Female Non-Carriers	Age	Education	MMSE
Psychosis | *p* = 0.023 OR = 4.15 95% CI [1.21, 14.2]	0.409	0.034	0.021
Female Heterozygotes vs. Female Non-Carriers			
Psychosis | *p* = 0.048 OR = 2.37 95% CI [1.01, 5.59]	0.287	0.350	0.016
Female Homozygotes vs. Female Heterozygotes			
Psychosis | *p* = 0.688 OR = 0.83 95% CI [0.33, 2.04]	0.322	0.747	<0.001
Males			
Male Homozygotes vs. Male Non-Carriers			
Psychosis | *p* = 0.995 OR = 1.01 95% CI [0.39, 2.57]	0.086	0.615	<0.001
Male Heterozygotes vs. Male Non-Carriers			
Psychosis | *p* = 0.299 OR = 1.46 95% CI [0.71, 3.00]	0.670	0.331	<0.001
Male Homozygotes vs. Male Heterozygotes			
Psychosis | *p* = 0.373 OR = 0.68 95% CI [0.301, 1.56]	0.718	0.647	<0.001
Between Sex			
Male Homozygotes vs. Female Homozygotes			
Psychosis | *p* = 0.160 OR = 2.20 95% CI [0.73, 6.61]	0.140	0.457	<0.001
Male Heterozygotes vs. Female Heterozygotes			
Psychosis | *p* = 0.148 OR = 1.60 95% CI [0.84, 3.05]	0.474	0.632	<0.001

**Table 3:** LB (−) Cohort, GAM.

Females	Covariates (*p*-value)		
Female Homozygotes vs. Female Non-Carriers	Age	Education	MMSE
Psychosis | *p* = 0.794 OR = 1.13 95% CI [0.42, 3.03]	0.441	0.310	0.036
Female Heterozygotes vs. Female Non-Carriers			
Psychosis | *p* = 0.301 OR = 0.71 95% CI [0.38, 1.34]	0.642	0.035	0.013
Female Homozygotes vs. Female Heterozygotes			
Psychosis | *p* = 0.388 OR = 1.55 95% CI [0.56, 4.28]	0.143	0.017	0.071
Males			
Male Homozygotes vs. Male Non-Carriers			
Psychosis | *p* = 0.209 OR = 1.75 95% CI [0.39, 2.57]	0.575	0.130	<0.001
Male Heterozygotes vs. Male Non-Carriers			
Psychosis | *p* = 0.328 OR = 1.38 95% CI [0.71, 3.00]	0.554	0.010	<0.001
Male Homozygotes vs. Male Heterozygotes			
Psychosis | *p* = 0.577 OR = 1.26 95% CI [0.301, 1.56]	0.291	<0.001	<0.001
Between Sex			
Male Homozygotes vs. Female Homozygotes			
Psychosis | *p* = 0.400 OR = 0.58 95% CI [0.16, 2.03]	0.659	0.080	<0.001
Male Heterozygotes vs. Female Heterozygotes			
Psychosis | *p* = 0.060 OR = 0.54 95% CI [0.29, 1.02]	0.648	<0.001	<0.001

## Data Availability

Data are available from the first author upon reasonable request.
