# Correction: Valcic et al. Determining Whether Sex and Zygosity Modulates the Association between APOE4 and Psychosis in a Neuropathologically-Confirmed Alzheimer’s Disease Cohort. *Brain Sci.* 2022, *12*, 1266

**DOI:** 10.3390/brainsci13010064

**Published:** 2022-12-29

**Authors:** Mila Valcic, Marc A. Khoury, Julia Kim, Luis Fornazzari, Nathan W. Churchill, Zahinoor Ismail, Vincenzo De Luca, Debby Tsuang, Tom A. Schweizer, David G. Munoz, Corinne E. Fischer

**Affiliations:** 1 Keenan Research Centre for Biomedical Science, Li Ka Shing Knowledge Institute, St. Michael’s Hospital, 209 Victoria Street, Toronto, ON M5B 1T8, Canada; 2 Department of Neurology, Faculty of Medicine, University of Toronto, Toronto, ON M5S 3H2, Canada; 3 Departments of Psychiatry, Clinical Neurosciences, Community Health Sciences, and Pathology, Hotchkiss Brain Institute and O’Brien Institute of Public Health, University of Calgary, Calgary, AB T2N 4Z6, Canada; 4 Division of Geriatric Psychiatry, Centre for Addiction & Mental Health, Toronto, ON M5T 1R8, Canada; 5 GRECC, VA Puget Sound and Department of Psychiatry and Behavioral Sciences, University of Washington, Seattle, WA 98195-6560, USA; 6 Institute of Medical Sciences, University of Toronto, Toronto, ON M5S 1A8, Canada; 7 Institute of Biomaterials and Biomedical Engineering, University of Toronto, Toronto, ON M5S 3G9, Canada; 8 Division of Neurosurgery, Department of Surgery, Faculty of Medicine, University of Toronto, Toronto, ON M5T 1P5, Canada; 9 Division of Neurosurgery, St. Michael’s Hospital, Toronto, ON M5B 1W8, Canada; 10 Department of Laboratory Medicine and Pathobiology, University of Toronto, Toronto, ON M5S 1A8, Canada; 11 Division of Pathology, St. Michael’s Hospital, Toronto, ON M5B 1W8, Canada; 12 Department of Psychiatry, Faculty of Medicine, University of Toronto, Toronto, ON M5T 1R8, Canada

## 1. Figure/Table Legend

In the original publication [1], there was an error in the location of the legend for Table 1. The legend was incorrectly placed under Figure 2. The correct legend appears below.

The legend for Table 1: Summary of descriptive statistics in neuropathologically confirmed AD patients, stratified by APOE4 status (E4 (−) = 0 APOE4 alleles, E4 = 1 APOE4 allele, E44 = 2 APOE4 alleles).

In the original publication, there was an error in the location of the legend for Figure 2. The legend was incorrectly placed under the legend for Table 1. The correct legend appears below. 

The legend for Figure 2: Graphical depiction of the average age of female APOE4 non-carriers and female APOE4 homozygotes, illustrating the significant difference between the two groups. On average, female APOE4 homozygotes were significantly younger than female APOE4 non-carriers (g = 0.42, 95%CI [0.05, 0.79], *p* = 0.03).

In the original publication, there was an error in the location of the legend for Table 2. The legend was incorrectly placed under the legend for Figure 3. The correct legend appears below.

The legend for Table 2: GAM Binary logistic regression analysis within the LB (+) cohort, examining the relationship between APOE4 status and the presence of psychosis across sex while adjusting for age, education and MMSE.

## 2. Text Correction

There was an error in the original publication. The NACC dataset that was utilized was incorrectly referred to as the Unified Data Set (UDS). The correct name for the dataset that was utilized is the Uniform Data Set (UDS).

A correction has been made to Section 2.1. Data Source: Two datasets were utilized: the Uniform Data Set (UDS) and the Neuropathology Data Set (NP).

## 3. Acknowledgments Correction

There was an error in the original publication. The paragraph indicated under Funding should be listed under Acknowledgments instead.

The following should be under Acknowledgments: The authors acknowledge NACC and the SMH Foundation Heather and Eric Donnelly endowment for their support of this project. The NACC database is funded by NIA/NIH Grant U24 AG072122. NACC data are contributed by the NIA-funded ADRCs: P30 AG062429 (PI James Brewer, MD, PhD), P30 AG066468 (PI Oscar Lopez, MD), P30 AG062421 (PI Bradley Hyman, MD, PhD), P30 AG066509 (PI Thomas Grabowski, MD), P30 AG066514 (PI Mary Sano, PhD), P30 AG066530 (PI Helena Chui, MD), P30 AG066507 (PI Marilyn Albert, PhD), P30 AG066444 (PI John Morris, MD), P30 AG066518 (PI Jeffrey Kaye, MD), P30 AG066512 (PI Thomas Wisniewski, MD), P30 AG066462 (PI Scott Small, MD), P30 AG072979 (PI David Wolk, MD), P30 AG072972 (PI Charles DeCarli, MD), P30 AG072976 (PI Andrew Saykin, PsyD), P30 AG072975 (PI David Bennett, MD), P30 AG072978 (PI Neil Kowall, MD), P30 AG072977 (PI Robert Vassar, PhD), P30 AG066519 (PI Frank LaFerla, PhD), P30 AG062677 (PI Ronald Petersen, MD, PhD), P30 AG079280 (PI Eric Reiman, MD), P30 AG062422 (PI Gil Rabinovici, MD), P30 AG066511 (PI Allan Levey, MD, PhD), P30 AG072946 (PI Linda Van Eldik, PhD), P30 AG062715 (PI Sanjay Asthana, MD, FRCP), P30 AG072973 (PI Russell Swerdlow, MD), P30 AG066506 (PI Todd Golde, MD, PhD), P30 AG066508 (PI Stephen Strittmatter, MD, PhD), P30 AG066515 (PI Victor Henderson, MD, MS), P30 AG072947 (PI Suzanne Craft, PhD), P30 AG072931 (PI Henry Paulson, MD, PhD), P30 AG066546 (PI Sudha Seshadri, MD), P20 AG068024 (PI Erik Roberson, MD, PhD), P20 AG068053 (PI Justin Miller, PhD), P20 AG068077 (PI Gary Rosenberg, MD), P20 AG068082 (PI Angela Jefferson, PhD), P30 AG072958 (PI Heather Whitson, MD), P30 AG072959 (PI James Leverenz, MD). Data from the Alzheimer’s Disease Genetic Consortium (ADGC) was used, NIA/NIH Grant U01 AG032984.

The authors state that the scientific conclusions are unaffected. This correction was approved by the Academic Editor. The original publication has also been updated.

## References

[1] Valcic M., Khoury M.A., Kim J., Fornazzari L., Churchill N.W., Ismail Z., De Luca V., Tsuang D., Schweizer T.A., Munoz D.G. (2022). Determining Whether Sex and Zygosity Modulates the Association between APOE4 and Psychosis in a Neuropathologically-Confirmed Alzheimer’s Disease Cohort. Brain Sci..

